# VIRMA promotes neuron apoptosis via inducing m6A methylation of STK10 in spinal cord injury animal models

**DOI:** 10.1111/cns.14453

**Published:** 2023-09-18

**Authors:** Hongxiang Hong, Guanhua Xu, Guofeng Bao, Jinlong Zhang, Chu Chen, Jiajia Chen, Chunshuai Wu, Jiawei Jiang, Jiayi Huang, Haiming Huang, Zhiming Cui

**Affiliations:** ^1^ Department of Spine Surgery The second Affiliated Hospital of Nantong University Nantong China; ^2^ Research Institute for Spine and Spinal Cord Disease of Nantong University Nantong China; ^3^ Department of Ultrasonography The Second Affiliated Hospital of Nantong University Nantong University Nantong China

**Keywords:** apoptosis, m6A methylation, spinal cord injury, STK10, VIRMA

## Abstract

**Background:**

Spinal cord injury (SCI) occurs as a devastating neuropathic disease. The role of serine–threonine kinase 10 (STK10) in the development of SCI remains unclear.

**Objective:**

This study aimed to investigate the action of m6A methylation on STK10 in the apoptosis of spinal cord neurons in the pathogenesis of SCI and the possible underlying mechanisms.

**Methods:**

Rat model of SCI was established and subsequently evaluated for motor function, pathological conditions, and apoptosis of spinal cord neurons. And the effects of overexpression of STK10 on neuronal cells in animal models of spinal cord injury and glyoxylate deprivation (OGD) cell models were evaluated. m6A2Target database and SRAMP database were used to predict the m6A methylation sites of STK10. The methylation kits were used to detect overall m6A methylation. Finally, the interaction between STK10 and vir like m6A methyltransferase associated (VIRMA) was explored in animal and cellular models.

**Results:**

STK10 is markedly decreased in spinal cord injury models and overexpression of STK10 inhibits neuronal apoptosis. VIRMA can induce m6A methylation of STK10. VIRMA is over‐expressed in spinal cord injury models and negatively regulates the expression of STK10. m6A methylation and apoptosis of neuronal cells are reduced by the knockdown of VIRMA and STK10 shRNA have shown the opposite effects.

**Conclusions:**

VIRMA promotes neuronal apoptosis in spinal cord injury by regulating STK10 m6A methylation.

## Graphical abstract

Decreased expression of STK10 in rats with spinal cord injury. Decreased expression of STK10 in rats with spinal cord injury. (A–F) Bioinformatic analysis of GSE132552 chip. (G) Results of H&E staining. (H) BBB score. (I) Results of TUNNEL staining. (J) STK10 mRNA expression by RT‐qPCR assay. (K) STK10 protein expression by WB assay. **p < 0.01, ***p < 0.001.

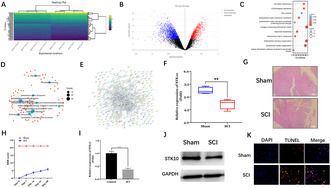

## 1 INTRODUCTION

Spinal cord injury (SCI) has been described as a disabling injury to the central nervous system that severely impacts the sensory and motor functions of patients.^1^ Impaired sensory, motor or autonomic function below the site of damage to the spinal cord is the primary symptom of SCI.^2^ On average, there are 236 to 1009 SCI occurrences per million people worldwide.^3^ In China, the prevalence of SCI is 37 per million people, with an average age of 34.7–54.4 years.^4^ Besides, SCI patients are vulnerable to complications regarding low heart rate, low blood pressure, impaired thermoregulation, and other symptoms like respiratory dysfunction.^5^, ^6^ However, the clinical efficacy of treatments including surgery, medication, and Chinese medicine remains insufficient, either for the injury of SCI itself or for the complications it causes.^7^ Additionally, the complex physiopathological variations in the SCI process pose a great challenge to the treatment after SCI.^8^

The main factors for the unsatisfactory results of SCI treatment are related to the difficulty of neuronal regeneration and the mechanism of injury.^9^ Injuries to the spinal cord are composed of primary and secondary injuries.^10^ Primary injury refers to damage caused by mechanical forces acting directly or indirectly on the spinal cord, which is usually irreversible, such as neuronal death.^11^ Apoptosis is highly detrimental to non‐regenerative neurons, and the extent and severity of apoptosis directly affect the prognosis of SCI.^12^ Therefore, the protection of neurons after spinal cord injury and the reduction of neural death are the keys to promoting neural regeneration and maximizing the recovery of spinal cord function.^13^ Many research hotspots in the field of SCI are currently investigating the factors influencing neuronal apoptosis and its mechanisms to explore the prognosis of spinal cord injury through the regulation of apoptosis and to develop new therapeutic approaches.^14^

N6‐methyl‐adenosine (m6A) methyltransferases were reported to participate in the pathogenesis of many diseases, for example, obesity, cardiovascular disease and cancers.^10^ Vir‐like m6A methyltransferase associated (VIRMA) is known as a writer for m6A methylation.^11^ Results of previous studies suggested that VIRMA may be involved in the development of many diseases.^14^ However, the effects of VIRMA on SCI have been scarcely reported.

A serine–threonine kinase 10 (STK10) is a member of the STE20 serine/threonine kinase family. Produced mainly in lymphoid organs such as the spleen, thymus, and bone marrow.^15^ STK10 participates in a variety of intracellular functions, like cell proliferation, apoptosis regulation, cytoskeletal rearrangement, and cell motility.^16^ At present, there are not many studies on STK10 in diseases. Leroy et al.^17^ noted that cleavage of STK10 during apoptosis eliminated its kinase activity, leading to reduced moesin phosphorylation. Also, Fukumura et al.^18^ demonstrated that STK10 may promote oncogenic effects by inhibiting apoptotic signaling in cancer through polymorphisms or somatic mutations. The latest study provided the first evidence that STK10 exerts an important role in the proliferation and migration of prostate cancer cells.^19^ However, STK10 has not been studied in SCI for the time being. Therefore, the present experiment proposed to investigate the possible interaction between VIRMA and STK10 in the development of SCI, as well as to explore the underlying possible mechanisms. We will examine the expression of STK10 in spinal cord injury models, and also explore the effects of overexpression of STK10 on neuronal apoptosis; furthermore, the effects of VIRMA on m6A methylation of STK10 will be examined, and whether VIRMA promotes neuronal apoptosis in spinal cord injury via regulating STK10 m6A methylation and the underlying mechanism will also be evaluated.

## 2 MATERIALS AND METHODS

### 2.1 Data collection and analysis

Genomic profiling datasets GSE45006 were downloaded from the Gene Expression Omnibus database (http://www‐ncbi‐nlm‐nih‐gov.proxy.library.carleton.ca/geo/). The Sequence‐based RNA Adenosine Methylation Sites Predictor (SRAMP, http://www.cuilab.cn/sramp/) database and m6A2Target (http://m6a2target.canceromics.org) database were applied to predict the m6A methylation sites.^20^, ^21^

### 2.2 Animals

A total of 35 specific pathogen‐free (SPF) adult male Sprague–Dawley rats obtained from the animal experimental department of Nantong University were housed in individual cages. All rats were acclimatized to their environment for at least 1 week before the experiment, with a 12‐h light–dark cycle, a temperature of 23 ± 1°C, relative humidity of 50%, and an ad libitum supply of food and water. The study was approved by the Animal Welfare Ethics Division of the second Affiliated Hospital of Nantong University Bioethics Committee.

### 2.3 Establishment and intervention of spinal cord injury in rats

Rats were anesthetized by intraperitoneal injection of 2% sodium pentobarbital (0.1 mL/kg). The dorsal skin of the rats was dissected to expose the spinous processes and laminae of T8‐10. A 10‐g hammer was smashed into the T9 portion of the spinal cord from a height of 25 mm, and then a large amount of pressure was applied to the spinal cord by hand for 1 min to injure the rats and then sutured. In the sham‐operated group, the incision was closed layer by layer right after exposing the spinal cord, and no injury was induced. The criteria for successful generation of the rat SCI model were contusions at the site of injury, twitching of both lower extremities, and spastic tail wagging.

### 2.4 Behavioral tests

The Basso–Beattie–Bresnahan (BBB) motor function score is a criterion for evaluating the motor function of the hind limbs of animals. Rats were placed on a circular platform with a diameter of 2 m and hind limb walking and limb activity scores were observed and recorded. The joint activity of the hind limbs of the animals was scored in subsections 0–7. The gait and coordination of the hind limbs were scored in subsections 8–13. The fine movements of the paws were judged in subsections 14–21. The scores of the three stages were summed to 21 points. Each group was scored 1 day before surgery and 1, 3, 7, 10, 14, 21, and 28 days post‐injury (dpi).

### 2.5 Hematoxylin–eosin staining (H&E staining)

Spinal cord tissue was removed after sacrificing the rats and embedded into paraffin to make 5‐μm sections. Hematoxylin staining was performed for 1 min and then rinsed twice in distilled water. Sections were divided into 1% hydrochloric acid and rinsed twice in distilled water. Continued staining with eosin (Sigma‐Aldrich) for 2 min.

### 2.6 Cell culture and treatment

PC12 cell lines (purchased from ATCC) were cultured in Dulbecco's modified Eagle's medium (DMEM, Gibco) supplemented with 10% fetal bovine serum (Gibco) and 1% penicillin/streptomycin (Gibco) in a 37°C incubator containing 5% CO_2_. The indicated overexpression plasmids or small interfering RNA (siRNA) were transfected into the culture medium using Lipofectamine 3000 (Invitrogen).

### 2.7 Construction of oxygen and glucose deprivation model

Cells were cultured with fetal bovine serum (FBS) and glucose‐free Dulbecco's modified Eagle's medium (DMEM) at 37°C for 6 h. After oxygen–glucose deprivation (OGD), the medium was replaced with a normal medium containing DMEM with high glucose, 10% FBS, and 1% penicillin/streptomycin for 24 h at 37°C in an incubator containing 5% CO_2_.

### 2.8 Lactate dehydrogenase assay

Lactate dehydrogenase (LDH) was measured using the LDH Assay Kit (Beyotime) to measure the release of LDH. The treated cell culture medium was incubated with LDH working reagent for 30 min and measured according to the manufacturer's instructions. Quantitative results were obtained with a spectrophotometer (Epoch) at 490 nm.

### 2.9 TdT‐mediated dUTP nick‐end labeling staining

TdT‐mediated dUTP nick‐end labeling (TUNEL) staining was performed following the instructions of the manufacturer. Samples were stained in FITC—12‐dUTP Labeling Mix (1 μg/mL), incubated for 5 min at room temperature in the dark, and then washed three times with PBS. Three randomly selected fields of view were observed and photographed under the inverted microscope (100×). The images were analyzed and counted with Image‐Pro 6.0 software. Positive cells had brownish‐yellow cytoplasm and blue nuclei. Apoptosis was determined as the ratio of positive cell count/total cell count × 100.

### 2.10 Quantification of N6‐methyladenosine levels

The m6A levels of total RNA were quantified with the Colorimetric EpiQuik m6A RNA Methylation Quantification Kit (Epigentek, #P‐9005). Briefly, approximately 200 ng of RNA is bound to each well utilizing RNA High Binding Solution, and m6A is collected and detected by the corresponding antibody. The OD of each sample was then detected at 450 nm using a microplate spectrophotometer. The percentage of m6A in total RNA was calculated as follows: m6A% = (sample OD − negative control OD) ÷ 200 ÷ (positive control OD − negative control OD) × 100%.

### 2.11 Dual luciferase reporter assay

Transfect cells with 500 ng WT or mutant pMIR‐VIRMA vector or control pMIR‐VIRMAV using Hieff Trans™ Liposome Transfection Reagent according to the manufacturer's instructions. Seventy‐two hours post‐transfection, cells were harvested and lysed. The collected supernatant was then assayed for luciferase activity using a dual luciferase reporter system (Promega).

### 2.12 N6‐methyladenosine‐RNA immunoprecipitation‐quantitative polymerase chain reaction

N6‐methyladenosine‐RNA immunoprecipitation‐quantitative polymerase chain reaction (MeRIP) is tested with the EpiQuik CUT&RUN m6A RNA Enrichment (MeRIP) kit. Briefly, the RNA sequence at both ends of the target m6A‐containing region is cleaved and the target m6A‐containing fragment is pulled down using a bead‐conjugated m6A capture antibody. The enriched RNA was then released, purified and eluted for Real‐time quantitative polymerase chain reaction (RT‐qPCR).

### 2.13 Real‐time quantitative polymerase chain reaction

Total RNA was extracted from spinal cord tissue or cells using TRIzol (Invitrogen, Thermo Fisher Scientific, Inc.). Then it was reverse transcribed into cDNA using FastKing cDNA First Strand Synthesis Kit (Tiangen). RT‐qPCR was performed with TB Green Premix Ex Taq II Kit (Takara Bio, #RR820A). Three replicate wells were set up for each reaction. The expression level of 3‐phosphoglycerate dehydrogenase (GAPDH) was used as an internal control. The sequences of the primers are shown below.

### 2.14 Western blotting

Equal amounts of denatured proteins are extracted from cells or tissue homogenates with lysates containing protease inhibitors. Protein concentration is measured with the bicinchoninic acid assay (BCA) assay kit. Proteins were electrophoresed on polyacrylamide gels and transferred to polyvinylidene difluoride membranes (PVDF, Millipore). Proteins were electrophoresed on polyacrylamide gels and transferred to polyvinylidene difluoride membranes (PVDF, Millipore). PVDF membranes were blocked for 2 h at room temperature using tris buffered saline with tween 20 (TBST) containing 5% skim milk. Incubate overnight at 4°C with the addition of primary antibodies. Secondary antibodies are then added and incubated for 2 h at 37°C. Proteins on PVDF membranes were quantified using an enhanced chemiluminescence system (ECL) and subsequently analyzed using Image Quant TL software (GE).

### 2.15 Statistical analysis

Data are expressed as mean (M) ± standard deviation (SD). Statistical analysis was performed by SPSS version 22.0. Two‐tailed unpaired Student's *t*‐test was applied for the comparison of the two data sets. Analysis of variance was used for the comparison among multiple groups. *p* Value < 0.5 was considered as significant differences.

## 3 RESULTS

### 3.1 Decreased expression of STK10 in rats with spinal cord injury

**FIGURE 1:**
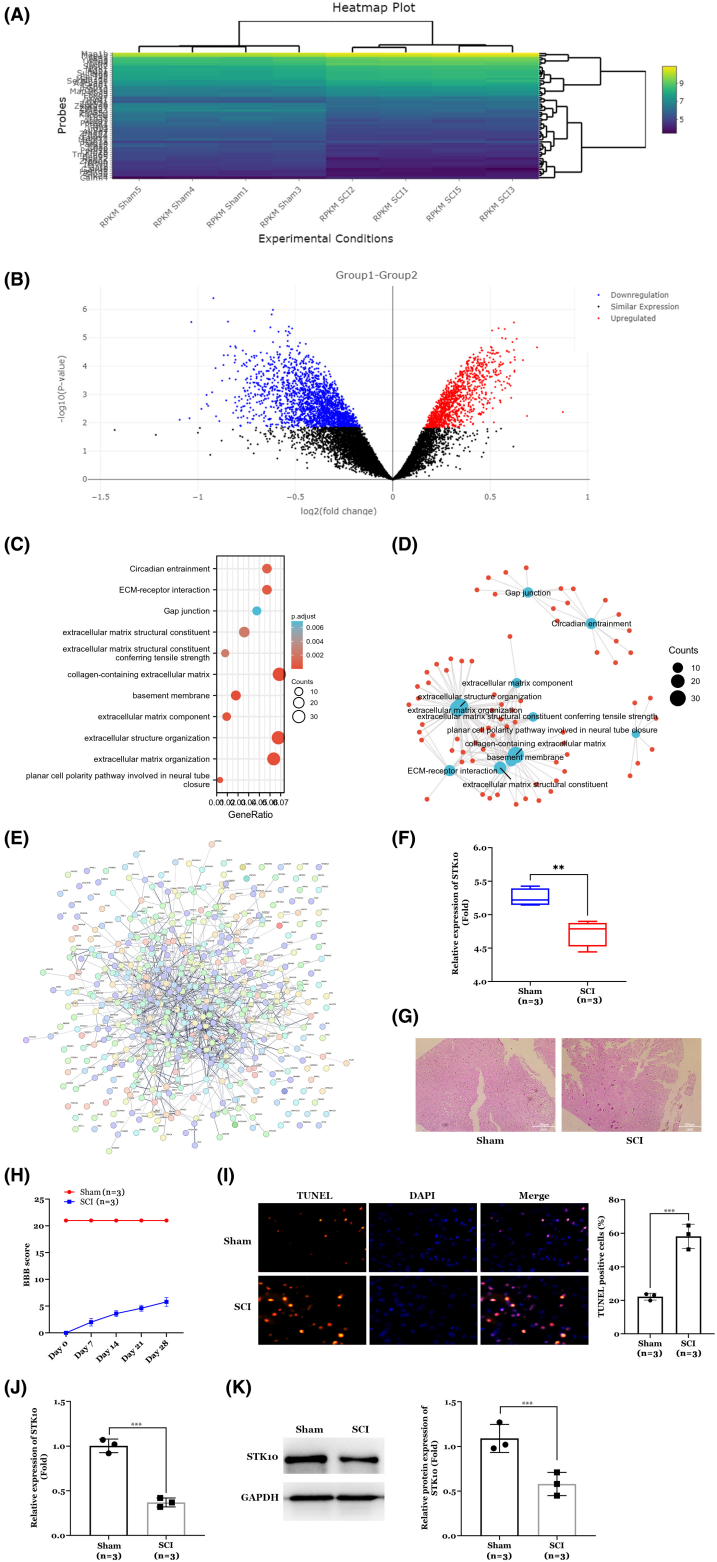
Decreased expression of STK10 in rats with spinal cord injury. (A–F) Bioinformatic analysis of GSE132552 chip. (G) Results of H&E staining. (H) BBB score. (I) Results of TUNNEL staining. (J) STK10 mRNA expression by RT‐qPCR assay. (K) STK10 protein expression by WB assay. **p < 0.01, ***p < 0.001.

First, we performed bioinformatic analysis of GSE132552 chip and compared gene expression difference between normal rats and spinal cord injury, and screened STK10 as one of the most significantly low expression genes (Figure 1A–F). Next, we established SCI rat model and results of H&E staining (Figure 1G) and BBB score (Figure 1H) and TUNNEL staining (Figure 1I) showed we successfully induced SCI condition in vivo. Furthermore, RT‐qPCR and WB analysis were performed for determining STK10 expression in SCI rats. We found that STK10 was markedly decreased in spinal cord of SCI rats on both mRNA and protein levels (Figure 1J,K).

### 3.2 Effects of STK10 on spinal cord injury animal model

**FIGURE 2:**
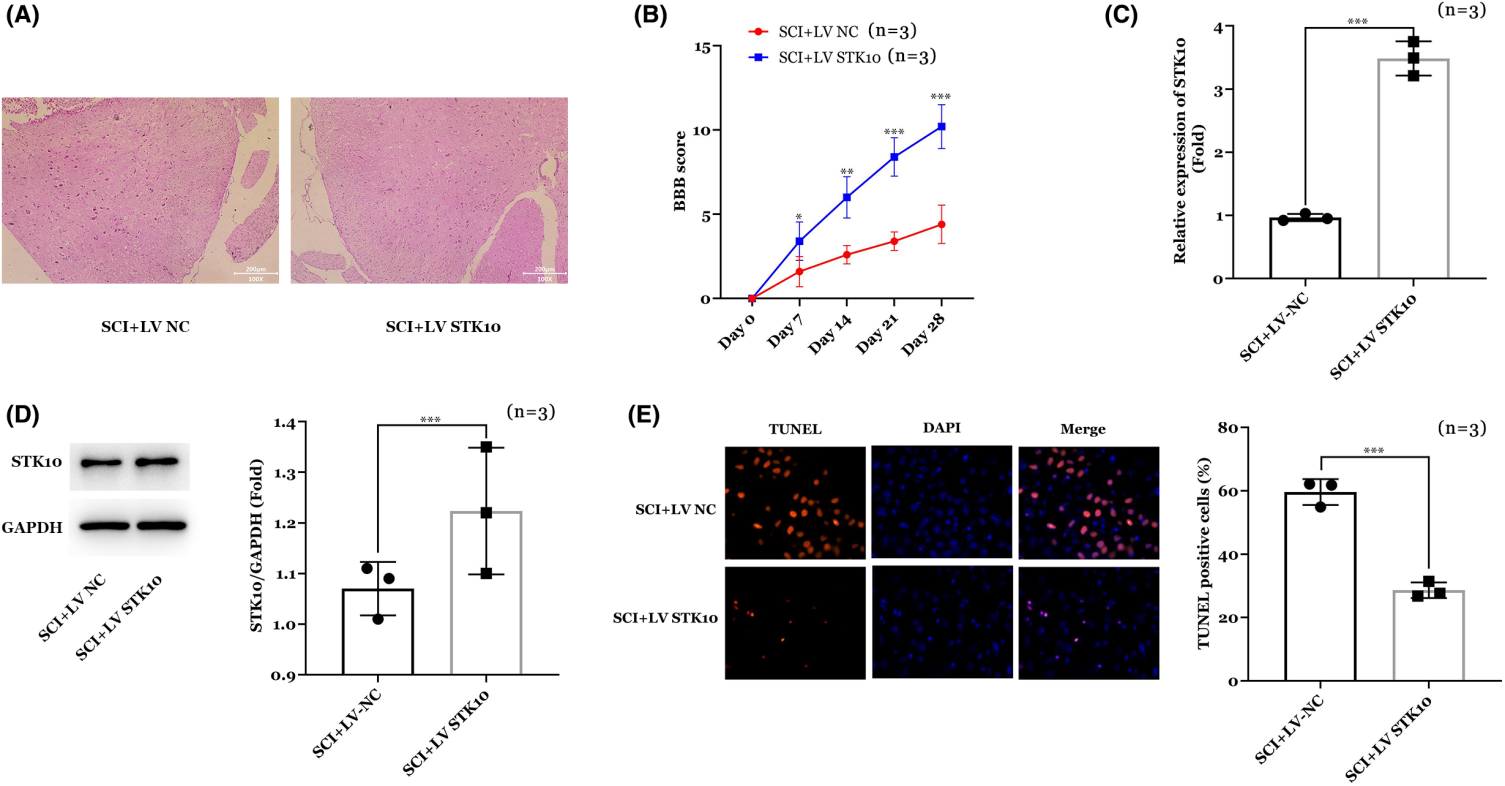
Effects of STK10 on spinal cord injury animal model. (A) Results of H&E staining. (B) BBB score. (C) STK10 mRNA expression by RT‐qPCR assay. (D) STK10 protein expression by WB assay. (E) Results of TUNNEL staining. *p < 0.05, **p < 0.01, ***p < 0.001.

Next, the rat model of spinal cord injury was established, and the recombinant lentivirus overexpression vector was injected through tail vein. The experiment was divided into two groups, SCI+ lentivirus vector negative control (LV NC) and SCI+ STK10 lentivirus vector (LV STK10) group. As shown in Figure 2, results of H&E staining (Figure 2A) and BBB score (Figure 2B) showed over‐expression of STK10 alleviated the SCI condition in vivo. Furthermore, PCR (Figure 2C) and WB (Figure 2D) analysis showed STK10 was markedly increased in LV STK10 compared with the SCI+ LV NC group on both mRNA and protein levels (Figure 2C,D, *p* < 0.001). Furthermore, results of TUNEL staining suggested over‐expression of STK10 inhibited the apoptosis of spinal cord in SCI rats in vivo (*p* < 0.001, Figure 2E).

### 3.3 Effect of STK10 on neuronal cell model of spinal cord injury

**FIGURE 3:**
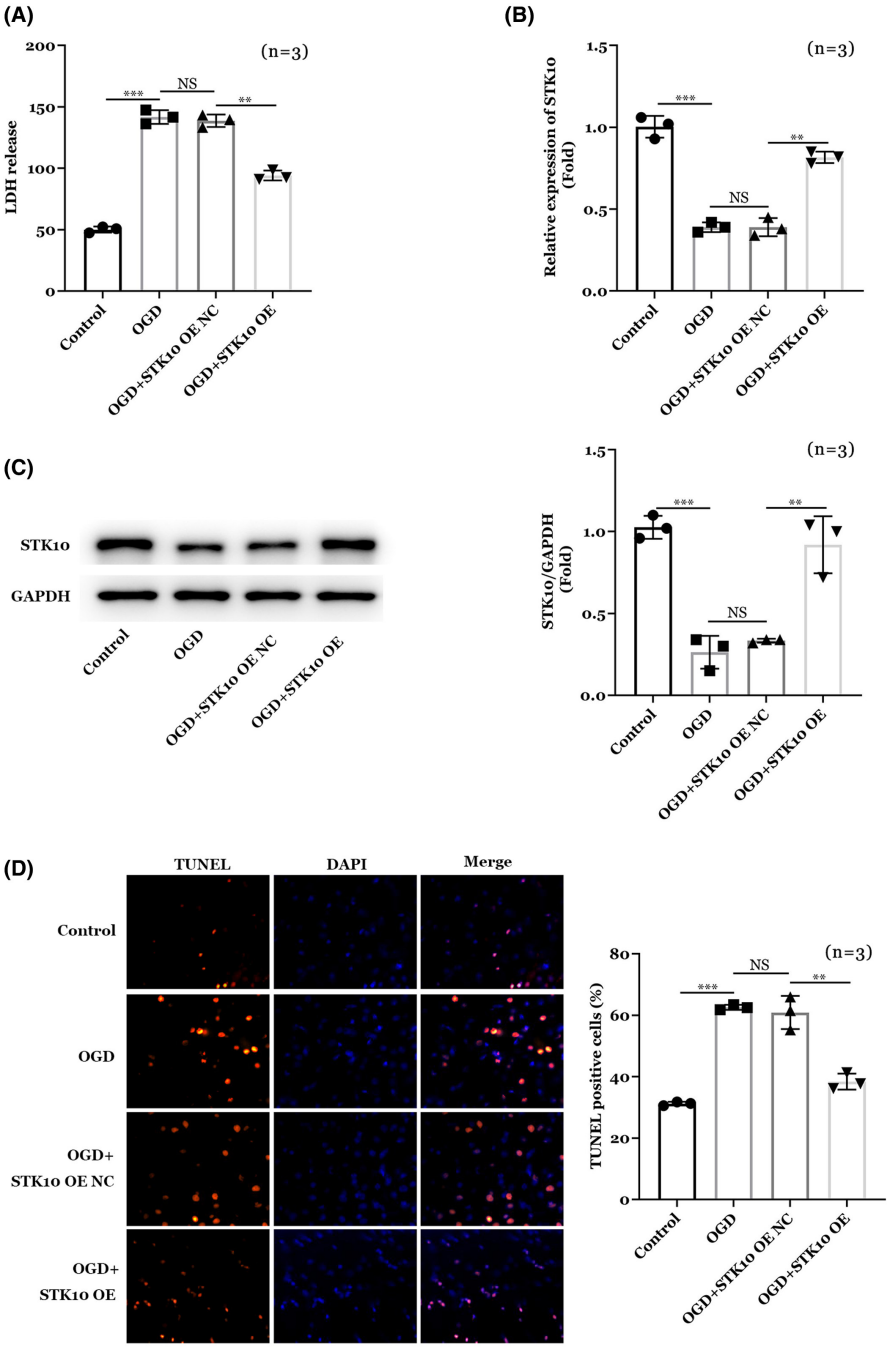
Effect of STK10 on neuronal cell model of spinal cord injury. (A) LDH release in neuron cells. (B) STK10 mRNA expression by RT‐qPCR assay. (C) STK10 protein expression by WB assay. (D) Results of TUNNEL staining. **p < 0.01, ***p < 0.001.

Next, neuron cells were cultured in vitro, and the spinal cord injury cell model was established by the method of glucose and oxygen deprivation (OGD), cells were divided into Control, OGD, OGD + STK10 OE NC, and OGD + STK10 OE groups. LDH release of cells in each group was detected to determine whether modeling is successful. As shown in Figure 3A, OGD treatment significantly increased LDH release in neuron cells, while STK10 OE markedly decreased LDH release in neuron cells (*p* < 0.01). These result suggested the in vitro spinal cord injury cell model was established. Moreover, we found OGD treatment decreased STK10 expression on both mRNA and protein levels and STK10 OE increased the expression of STK10 (Figure 3B,C). Furthermore, results of TUNNEL staining (Figure 3D) showed OGD treatment increased the apoptosis of neuron cells and overexpression of STK10 could partially alleviate the pro‐apoptotic effects of OGD treatment.

### 3.4 VIRMA can cause m6A methylation of STK10 in spinal cord injury

**FIGURE 4:**
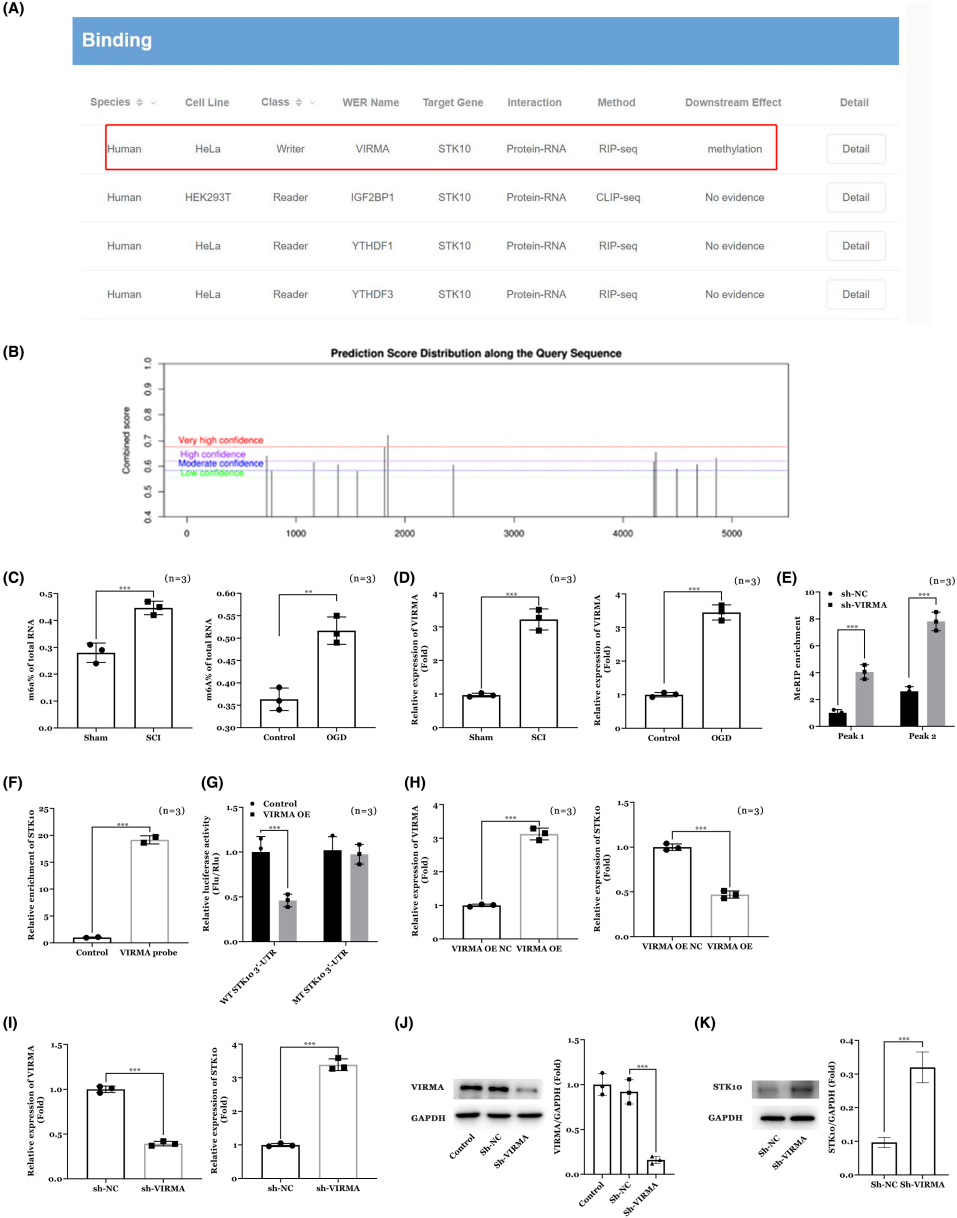
VIRMA can cause m6A methylation of STK10 in spinal cord injury. (A) Bioinformatic analysis of M6A2Target database. (B) Bioinformatic analysis of SRAMP database. (C) Overall m6A methylation in animal and cell models. (D) RT‐qPCR for VIRMA expression in SCI animal models and OGD neuron cells. (E) Results of Me‐RIP assay. (F) Results of RNA pull down assay. (G) Results of dual‐luciferase reporter assay. (H) Effects of VIRMA OE on VIRMA and STK10 mRNA expression. (I–K) Effects of VIRMA shRNA on VIRMA and STK10 mRNA and protein expression. **p < 0.01, ***p < 0.001.

Bioinformatic analysis of M6A2Target database (Figure 4A) and SRAMP database (Figure 4B) analysis speculated that VIRMA can cause m6A methylation of STK10. Next, detection of the overall m6A methylation in animal and cell models were conducted by methylation kit. As shown in Figure 4C, m6A methylation was increased in both SCI animal models and OGD neuron cells (*p* < 0.01). Moreover, result of RT‐qPCR results showed that VIRMA was increased in both SCI animal models and OGD neuron cells (Figure 4D). Furthermore, results of Me‐RIP assay (Figure 4E), RNA pull down assay (Figure 4F) and dual‐luciferase reporter assay (Figure 4G) confirmed the interaction between VIRMA and STK10. Finally, over‐expression of VIRMA increased the expression of VIRMA and decreased the expression of STK10 (Figure 4H) while over‐expression of VIRMA decreased expression of STK10on both mRNA (Figure 4I) and protein levels (Figure 4J,K).

### 3.5 Effect of VIRMA on spinal cord injury animal model by regulating STK10 m6A methylation

**FIGURE 5:**
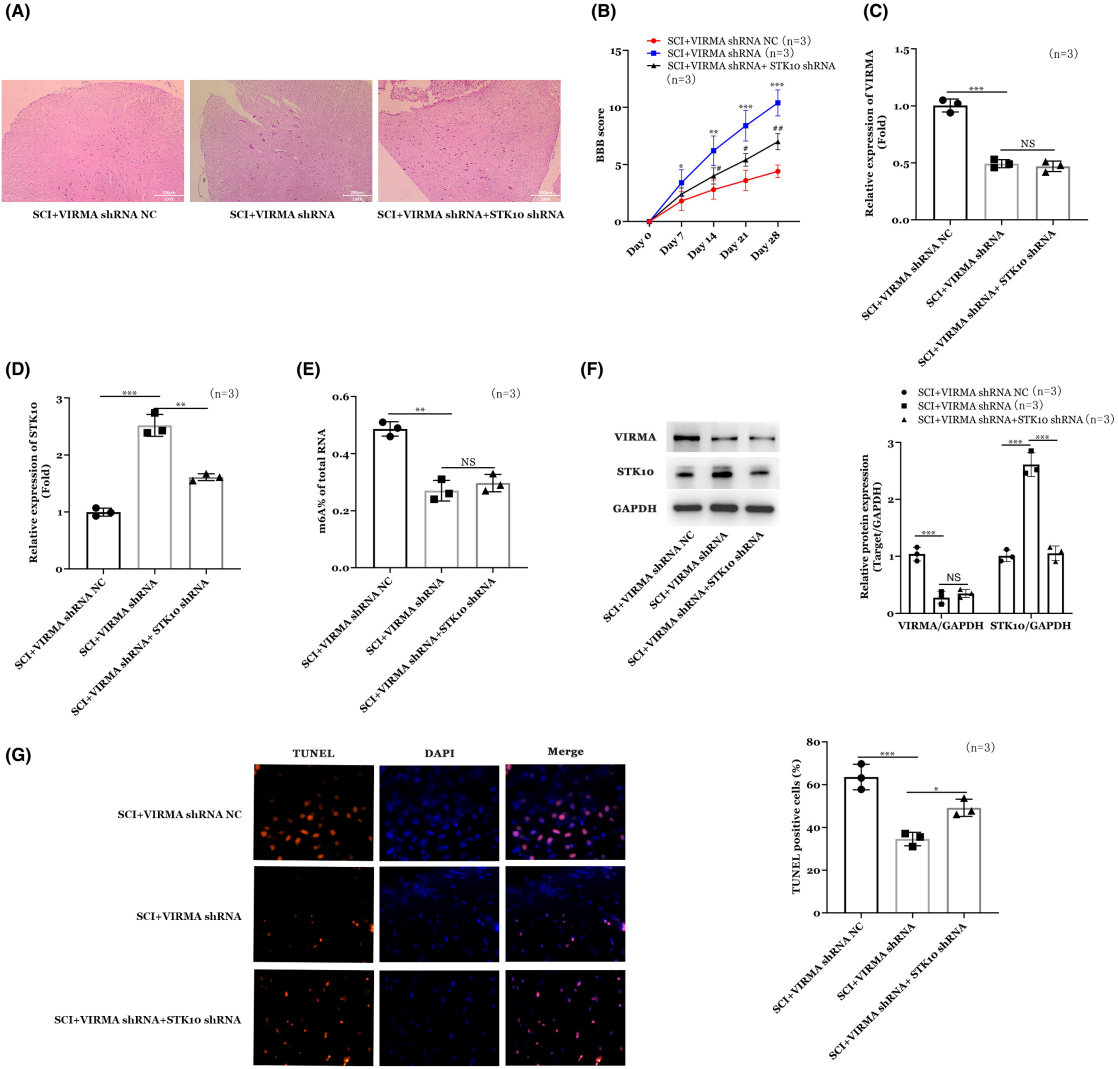
Effects of VIRMA on spinal cord injury animal model by regulating STK10 m6A methylation. (A) Results of H&E staining. (B) BBB score. (C) VIRMA expression by RT‐qPCR methods. (D) STK10 expression by RT‐qPCR methods. (E) level of m6A methylation. (F) WB results for VIRMA and STK10 expression. (G) TUNNEL staining results. *p < 0.05, **p < 0.01, ***p < 0.001.

The rat model of spinal cord injury was established. The recombinant lentivirus overexpression vector was injected through tail vein. The experiment was divided into three groups, namely, SCI + VIRMA shRNA NC, SCI + VIRMA shRNA, and SCI + VIRMA shRNA + STK10 shRNA groups. As shown in Figure 5A, VIRMA shRNA alleviated the spinal cord injury condition in rat models while STK10 shRNA partially alleviated the effects of STK10 shRNA (Figure 5A,B). Moreover, RT‐qPCR (Figure 5C,D) and WB (Figure 5F) showed VIRMA shRNA increased the expression of STK10 in rat models, while the addition of STK10 shRNA in VIRMA shRNA treated rats decreased STK10 expression. Next, detection of overall m6A methylation in animal models by methylation kit and we found that VIRMA shRNA decreased the level of m6A methylation in animal models (Figure 5E). Finally, results of TUNNEL staining showed VIRMA shRNA decreased the apoptosis in spinal cord injury animals and STK10 shRNA partially abrogated the effects of VIRMA shRNA (Figure 5G).

### 3.6 Effects of overexpression of STK10 on neuronal cell spinal cord injury model

**FIGURE 6:**
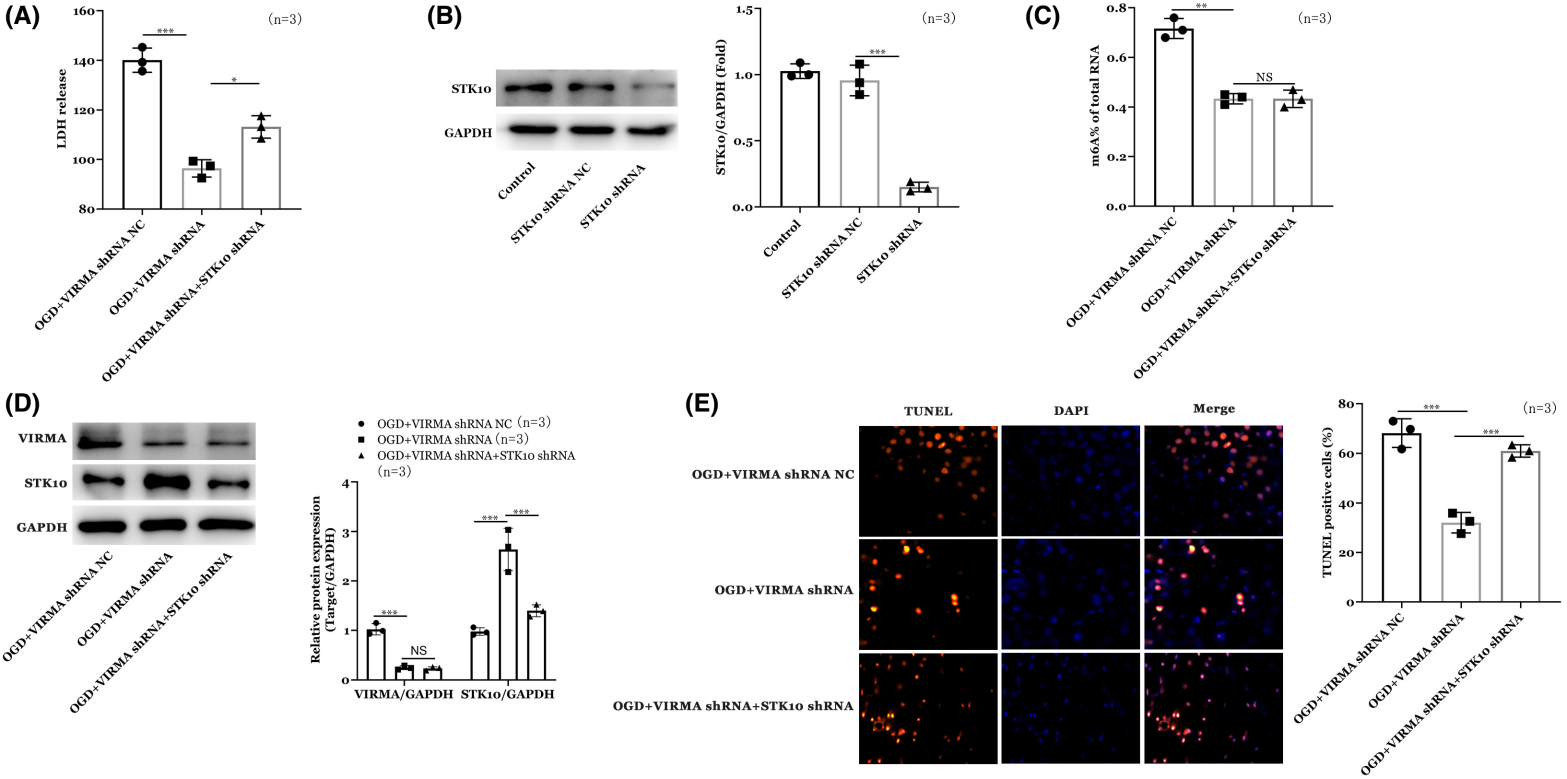
Effects of overexpression of STK10 on neuronal cell spinal cord injury model. (A) LDH release. (B) WB results for STK10 expression. (C) Overall m6A methylation in cell model. (D) WB results for VIRMA and STK10 expressions. (E) TUNNEL staining results. *p < 0.05, **p < 0.01, ***p < 0.001.

Finally, neurons were cultured in vitro, and the spinal cord injury cell model was established by glucose and oxygen deprivation (OGD). They were divided into OGD + VIRMA shRNA NC, OGD + VIRMA shRNA, and OGD + VIRMA shRNA+STK10 shRNA groups. Results of LDH release confirmed the establishment of the spinal cord injury model (Figure 6A). Moreover, WB results showed STK10 shRNA decreased STK10 expression in PC12 cells (Figure 6B). Next, detection of overall m6A methylation in cell model was conducted by methylation kit. We found OGD increased overall m6A methylation of the cells and VIRMA shRNA decreased the m6A methylation of the cells (Figure 6C). Furthermore, WB results showed VIRMA shRNA decreased VIRMA and increased STK10 expression in OGD treated cells, while co‐transfection of STK10 shRNA partially abrogated the effects of VIRMA shRNA (Figure 6D). Finally, TUNNEL staining results showed OGD decreased the apoptosis of the neurons and STK10 shRNA partially abrogated the effects of VIRMA shRNA (Figure 6E).

## 4 DISCUSSION

The study demonstrates the role of VIRMA in the development of spinal cord injury and may act through the regulation of STK10 m6A methylation to promote neuronal apoptosis.

Previous studies have shown that the apoptosis of neurons appears to be particularly important in the development of SCI.^22^, ^23^ Thus, this is the breakthrough that we are going to explore outside of this study. Firstly, we analyzed the GSE45006 microarray to compare the gene expression differences in normal rats and spinal cord injury, and filtered out STK10 as one of the most significantly under‐expressed genes. It was verified after the successful establishment of the rat spinal cord injury model that STK10 was abnormally low expressed in the rat spinal cord injury model. Moreover, overexpression of STK10 effectively enhanced motility and reduced apoptosis in SCI rats. Subsequently, we constructed neuronal cell OGD models in vitro for an in‐depth investigation. Similarly, STK10 expression was reduced in OGD‐treated cells, and apoptosis was reduced after overexpression of STK10. Specifically, STK10 overexpression inhibited apoptosis in spinal cord neuronal cells.

N6 methyladenosine (m6A) one of the most common (over 60%) RNA epigenetic modifications that add a methyl group to the N atom at position 6 on mRNA, lncRNA adenine (A).^24^ Mounting evidence linking altered m6A RNA methylation levels to neurodevelopmental and central nervous system (CNS) dysfunction.^25^, ^26^ Xing et al.^27^ revealed that the m6ARNA methylation profile was altered after SCI in zebrafish and that methyltransferase‐like 3 may be important for spinal cord regeneration. Based on the fact that the m6A RNA methylation profile was altered in SCI, we further pondered this possible mechanism. The SRAMP database predicted that VIRMA leads to m6A methylation of STK10. We detected that overall m6A methylation was elevated and VIRMA was highly expressed in animal and cellular models. Moreover, STK10 expression was reduced after overexpression of VIRMA and increased after the knockdown of VIRMA. Dual luciferase reporter assay and Me‐RIP assay demonstrated that VIRMA negatively regulates STK10.

Then, STK10 and VIRMA were silenced to explore the effect of VIRMA‐mediated STK10 m6A methylation. STK10 was downregulated in OGD‐treated neurons and SCI rats after silencing VIRMA and STK10. The total m6A level showed no significant difference after silencing VIRMA and STK10. In vitro experiments demonstrated that neuronal viability was inhibited, and LDH release and apoptosis were promoted after silencing VIRMA and STK10. In vivo experiments showed decreased BBB locomotor scores of SCI rats, exacerbated pathological conditions, decreased motoneurons in the anterior horn, and increased apoptosis and inflammation after silencing VIRMA and STK10. Overall, VIRMA promoted spinal cord neuron apoptosis in SCI via mediating STK10 m6A methylation.

In conclusion, silencing VIRMA suppressed spinal cord neuron apoptosis in SCI via mediating STK10 m6A methylation, and thus providing novel insights into the management of SCI. Despite the findings, this study has some limitations. The downstream targets of VIRMA and other possible pathways were not investigated in this study. We will perform in‐depth analysis in the future to enrich and support this study.

## FUNDING INFORMATION

The research was supported by Project of Nantong Municipal Health Commission and Project of Nantong First People's Hospital (MS2022016, YPYJJZD008); Science and Technology Project of Nantong City (MS22022004, JC22022067, KD2022KYCXTD011); Project of Jiangsu Administration of Traditional Chinese Medicine (MS2022090); Postgraduate Research & Practice Innovation Program of Jiangsu Province (KYCX21_3107); Special Key Project of Clinical Basic Research of Nantong University (2022JZ004); Nantong Young Medical Experts Training Project (2023, No. 07).

## CONFLICT OF INTEREST STATEMENT

The authors declare no conflict of interest.

## Data Availability

The data that support the findings of this study are available on request from the corresponding author. The data are not publicly available due to privacy or ethical restrictions.
